# Encapsulation of brewing yeast in alginate/chitosan matrix: lab-scale optimization of lager beer fermentation

**DOI:** 10.1080/13102818.2014.910373

**Published:** 2014-07-08

**Authors:** Vessela Naydenova, Mariyana Badova, Stoyan Vassilev, Vasil Iliev, Maria Kaneva, Georgi Kostov

**Affiliations:** ^a^ Department of Wine and Brewing Technology, University of Food Technologies, Plovdiv, Bulgaria

**Keywords:** encapsulation, lager beer fermentation, optimization, productivity

## Abstract

Two mathematical models were developed for studying the effect of main fermentation temperature (*T*_MF_), immobilized cell mass (*M*_IC_) and original wort extract (OE) on beer fermentation with alginate-chitosan microcapsules with a liquid core. During the experiments, the investigated parameters were varied in order to find the optimal conditions for beer fermentation with immobilized cells. The basic beer characteristics, i.e. extract, ethanol, biomass concentration, pH and colour, as well as the concentration of aldehydes and vicinal diketones, were measured. The results suggested that the process parameters represented a powerful tool in controlling the fermentation time. Subsequently, the optimized process parameters were used to produce beer in laboratory batch fermentation. The system productivity was also investigated and the data were used for the development of another mathematical model.

## Introduction

Fermentation is an important field of interest for system engineering due to its complex biological non-linear phenomena and dynamic processes.[1] The fermentation dynamics are affected by the raw material quality, the temperature and the biomass concentration. Therefore, mathematical model development is an important step towards the determination of suitable fermentation parameters in order to achieve an optimized process.[1–4]

In recent years, an important branch of biotechnology has been devoted to the development of proper fermentation processes and efficient steps in the utilization of fermentation technology.[1] A lot of research has been focused on the immobilization of yeast cells and their application in brewing. The main advantages of using immobilized cells for beer production are enhanced fermentation productivity due to higher biomass densities, improved cell stability, easier implementation of continuous operation, improved operational control and flexibility, facilitated cell recovery and reuse, and simplified downstream processing (for review see [5]). The intensification of a particular fermentation process using immobilized cell technology (ICT) is generally industrialized if the new characteristics acquired result in a more economical system, and the new technology can be readily scaled up.[5] However, every step taken in cost reduction should preserve the product flavour.[6] ICT processes have been designed for different stages in beer fermentation, including wort acidification, bioflavouring during the secondary fermentation, primary fermentation and fermentations for the production of alcohol-free or low-alcohol beers.[7] Key parameters of this technology are the selection of carrier material and immobilization method together with bioreactor design. The determination of these parameters is governed by operational conditions such as temperature, pH, substrate composition and fluid dynamics.[5,8] Therefore, a major challenge for the successful industrial-scale application of ICT is the flavour profile control during combined primary and secondary fermentation.[9] At present, only beer maturation and alcohol-free beer production are obtained by means of commercial-scale immobilized yeast reactors.[10]

The aim of this work was to develop mathematical models of wort fermentation with immobilized cells for optimization of operational conditions (main fermentation temperature, original wort extract and immobilized cell mass) and process control. The main and secondary fermentation models were developed on the basis of laboratory data on fermentation dynamics. Once obtained, the primary fermentation model was used to get an optimal fermentation profile for beer production under laboratory conditions. Meanwhile, the system productivity model was determined, which could be used for the batch fermentation transfer into continuous mode.

## Materials and methods

### Microorganisms

The experiments were carried out with commercial dry brewing yeast strain *Saccharomyces pastorianus* (*carlsbergensis*) Saflager S-23 purchased from Fermentis (France).

### Cell immobilization

The yeast suspension was added to 100 cm^3^ of sodium alginate solution (30 g dm^−3^) and subsequently dropped into 100 cm^3^ of 20 g dm^−3^ CaCl_2_ solution. The cell concentration in the beads was 10^7^ CFU cm^−3^ of gel. The beads were left for 30 min in CaCl_2_ and were then placed into 100 cm^3^ of 3.8 g dm^−3^ chitosan solution in 1 cm^3^ × 100 cm^−3^ acetic acid. The alginate beads stayed in the chitosan solution for 60 min. Afterwards, the chitosan-alginate beads were washed with sterile water in order to remove the excess chitosan. The beads stayed in a 0.05 mol dm^−3^ sodium citrate solution for 30 min to obtain microcapsules with liquid core.[11–15]

### Wort

Wort with an original gravity (OG) of 17±0.5 °P was supplied by Kamenitza Plc. It was diluted to OG – 8.5±0.5, 10.5±0.5, 13±0.5, 15.5±0.5 and 17.5±0.5 °Р. All wort types were autoclaved at 120 °C for 20 min.

### Design of experiments and fermentation

Central composite design (CCD) of type 2^3^ with star arm and block structure was used for the optimization of the fermentation parameters main fermentation temperature (*T*_MF_), original wort extract (OE) and immobilized cell mass (*M*_IC_). The mathematical processing of the experimental results was done using Microsoft Excel and Statgraphics Centurion XV (trial version). The parameter values were pre-coded and the calculations were made according to the dependences shown in [16].

The fermentations (main and secondary) were carried out with 400 cm^3^ sterile wort in fermentation bottles equipped with airlocks. *T*_MF_, OE and *M*_IC_ were determined according to Table 2. The maturation temperature was 4 ºC higher than the *T*_MF_. Maturation started when the difference between the attenuation limit and apparent attenuation was approximately 20%.

**Table 1.** Limits of variation of fermentation parameters.

Factor	−α = −1.78885	−1	0	1	+α = 1.78885
*T*_MF_ (°С)	8.03	10	12.5	15	16.97
OE (°Р)	8.53	10.5	13	15.5	17.47
*М*_IC_ (g)	1.06	5	10	15	18.94

Note: T_MF_: main fermentation temperature; OE: original extract; *M*_IC_: immobilized cell mass

**Table 2.** Experimental design and results for the modelling and optimization of fermentation parameters.

		Coded values						*Q*_ETH_ (g dm^−3^ h^−1^)		*Q* (g dm^−3^ h^−1^)	
No.	Block	*T*_MF_	OE	*M*_IC_	Time of main fermentation (h)	Maturation time (h)	Fermentation time (h)	MF	F	*Q*_MF_	*Q*_F_
1	2	−1.78885	0	0	***636***	***50***	***686***	***0***.***08***	***0***.***08***	***5***.***78***	***8***.***26***
2	1	−1	−1	−1	***288***	***168***	***456***	***0***.***18***	***0***.***25***	***9***.***26***	***9***.***60***
3	1	−1	1	−1	***324***	***180***	***504***	***0***.***18***	***0***.***29***	***15***.***97***	***27***.***21***
4	1	−1	−1	1	***192***	***264***	***456***	***0***.***27***	***0***.***36***	***13***.***41***	***14***.***10***
5	1	−1	1	1	***300***	***204***	***504***	***0***.***20***	***0***.***33***	***17***.***49***	***28***.***72***
6	1	0	0	0	***204***	***180***	***384***	***0***.***27***	***0***.***40***	***18***.***76***	***22***.***47***
7	1	0	0	0	***196***	***172***	***368***	***0***.***29***	***0***.***42***	***19***.***53***	***23***.***38***
8	2	0	0	0	***200***	***190***	***390***	***0***.***28***	***0***.***41***	***19***.***14***	***22***.***91***
9	2	0	−1.78885	0	***108***	***156***	***264***	***0***.***39***	***0***.***54***	***11***.***51***	***13***.***41***
10	2	0	1.78885	0	***264***	***84***	***348***	***0***.***30***	***0***.***34***	***45***.***02***	***49***.***24***
11	2	0	0	−1.78885	***252***	***132***	***384***	***0***.***24***	***0***.***32***	***18***.***61***	***19***.***55***
12	2	0	0	1.78885	***96***	***168***	***264***	***0***.***60***	***0***.***90***	***40***.***49***	***49***.***94***
13	2	0	0	0	***210***	***175***	***385***	***0***.***27***	***0***.***39***	***18***.***22***	***21***.***82***
14	1	1	−1	−1	***144***	***96***	***240***	***0***.***31***	***0***.***47***	***14***.***16***	***19***.***34***
15	1	1	1	−1	***192***	***96***	***288***	***0***.***35***	***0***.***50***	***32***.***65***	***47***.***82***
16	1	1	−1	1	***78***	***126***	***204***	***0***.***67***	***0***.***89***	***36***.***50***	***33***.***87***
17	1	1	1	1	***120***	***168***	***288***	***0***.***59***	***0***.***83***	***65***.***02***	***69***.***61***
18	2	1.78885	0	0	***120***	***72***	***192***	***0***.***51***	***0***.***71***	***40***.***54***	***42***.***37***

Note: *T*_MF_: temperature of main fermentation; OE: original extract; *M*_IC_: immobilized cell mass; *Q*_ETH_: ethanol productivity; *Q*: beer productivity; MF: for main fermentation; F: for total fermentation time. Text in bold italics fonts represents the results for the objective functions.

### Productivity

The system productivity was calculated according to [17].

### Analytical methods

The characterization of wort, green beer and beer (original extract, degree of attenuation, extract, alcohol, pH, colour and vicinal diketones) was conducted according to the current methods recommended by the European Brewery Convention.[18] The detailed information on biomass concentration analysis can be found in [14]. The aldehyde concentrations were determined according to [19].

## Results and discussion

In our previous studies,[11–15] the fermentation dynamics were found to vary significantly depending on the following parameters: *T*_MF_ (respectively, the maturation temperature (*T*_MAT_)), *M*_IC_ and OE. Therefore, it can be suggested that the combination of these factors increases their influence on the fermentation dynamics.

**Figure 1.**
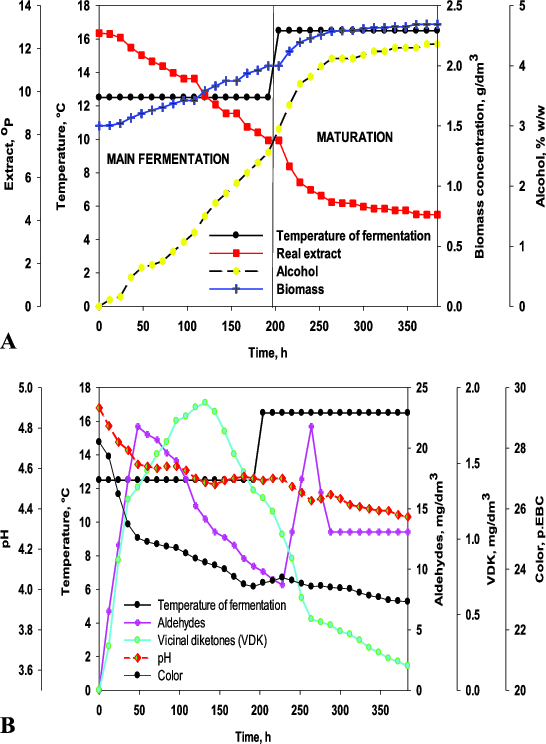
Fermentation dynamics (variant 6, Table 1): extract, ethanol, biomass concentration (**A**); carbonyl compounds, pH and colour of beer (**B**).

**Figure 2.**
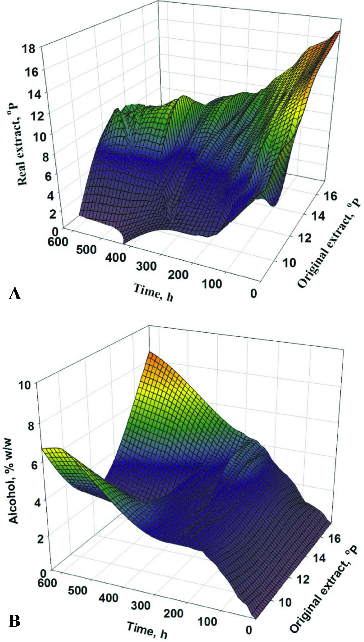
Dynamics of changes in extract (**A**) and alcohol (**B**) in the factor space for the time of fermentation.

**Figure 3.**
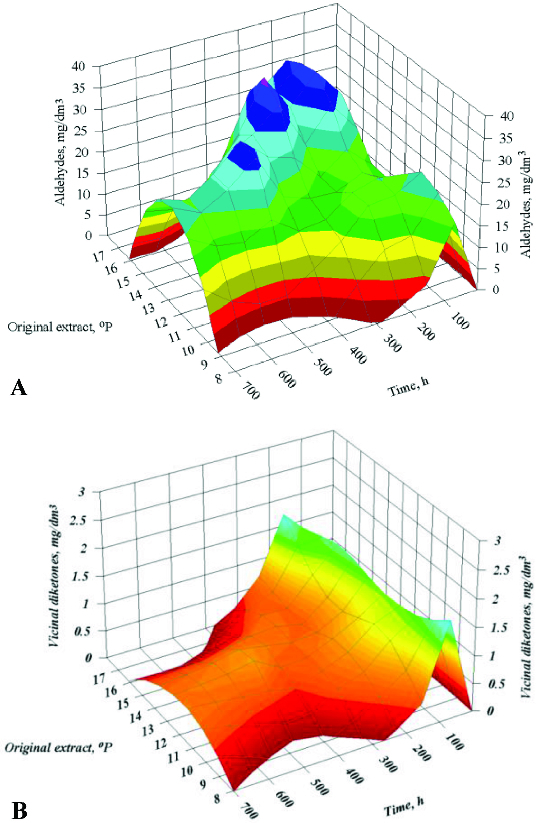
Dynamics of changes in carbonyl compounds in the factor space for the time of fermentation: aldehydes (**A**) and vicinal diketones (**B**).

Table 1 presents the limits of variation of *T*_MF_, *M*_IC_ and OE. Table 2 shows the results on the objective functions for optimization: primary fermentation time (MF) and maturation time (Mat*F*) for 18 fermentations, according to the planned experiment schedule. Table 2 also presents the system productivity data. Figure 1 shows the fermentation dynamics of one of the variants according to Table 2. The data on the other variants are summarized in three-dimensional graphics (Figures 2 and  3).

The dynamics of carbonyl compounds was investigated because the maturation time depends on the diacetyl concentration in beer. The increase in the fermentation temperature, the amount of immobilized cells and the initial wort extract resulted in increased concentrations of carbonyl compounds.[12,13,15] The higher alcohol and ester production was also affected by the factors studied, but unlike carbonyl compounds, alcohol and ester concentrations had no effect on maturation time. Therefore, these yeast metabolites were not an object of our investigations.

### Modelling of main fermentation time

**Figure 4.**
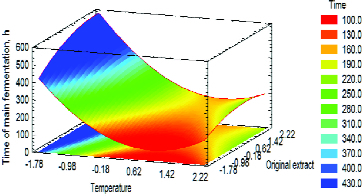
Estimated response surface for the influence of *T*_MF_ (A) and OE (B) on ‘Time of main fermentation’ at *M*_IC_ = 0 (10 g). Factors were presented with coded values.

Table S1 (see the Online Supplemental Appendix) and Figure 4 show the statistical analysis results on the influence of *T*_MF_, *M*_IC_ and OE on the main fermentation time. The non-significant variables were eliminated according to their *p*-value at a confidence level α = 0.95 (see Table S1 in Supplementary Material). The following adequate mathematical model was obtained after the non-significant variables were removed:(1) 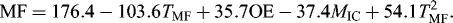

The model obtained (Equation 1) did not confirm the suggestion that the combined effect of the factors would be of key importance to the objective function. The main fermentation time decreased with the increase in *T*_MF_ and *M*_IC_. On the contrary, the OE increase resulted in prolonged primary fermentation. It can be assumed that the discrepant effect of *T*_MF_ and OE on the main fermentation time was due to the mass transfer in the capsule. Interestingly, there was a significant area in the response surface where the main fermentation time was up to 100 h (Figure 4). Therefore, the optimal values of the main fermentation time were observed between 48 h and 72 h.

The model parameters obtained resulted from the observed fermentation dynamics (Table 2). The lowest fermentation temperature (8 °C) led to prolonged main fermentation time (600 h). The secondary metabolite synthesis was suppressed and the maximum metabolite concentrations were observed at the end of the primary fermentation. The carbonyl compounds reduction was slow, which resulted in prolonged maturation.

At a constant *T*_MF_, *M*_IC_ affected the main fermentation time but did not affect the total fermentation time. The increase in *M*_IC_ accelerated the synthesis of secondary metabolites, which led to prolonged maturation. For example, at 10 °C, OE = 10.5 °P and *M*_IC_ = 5g, the main fermentation lasted longer than the fermentation carried out with 15 g immobilized cells, all other conditions being the same. The maximum aldehyde concentration was observed between 84 h and 180 h at a low *M*_IC_, while the increase in *M*_IC_ led to a distinct aldehyde maximum between 120 h and 144 h. The higher *M*_IC_ resulted in a double increase in the vicinal diketones concentration during the main fermentation. The higher vicinal diketones concentration was the main reason for the increase in maturation time by four days. The substitution of 10.5 °P wort by 15.5 °P wort did not change the observed trends.

The most interesting results were recorded during fermentation at 12.5 °C, *M*_IC_ = 1.05 g and OE = 13 °P. The microcapsules broke down rapidly due to the biomass growth, the cells began to leak in the medium and the fermentation could be regarded as a free cell process. The low *M*_IC_ led to the synthesis of low amounts of secondary metabolites which caused a reduction in maturation time. The *M*_IC_ increase to 10 g (the variants in the centre of the planned experiments) resulted in shorter main fermentation time. Nevertheless, the threefold increase in the vicinal diketones concentration compared to the previous variant determined prolonged maturation. The aldehydes peak was lower, but it was shifted to an earlier stage of the main fermentation. The almost double increase in *M*_IC_ to 18.94 g resulted in a 2- to 2.5-fold faster main fermentation. On the other hand, the accelerated fermentation accounted for 1.5-fold higher vicinal diketones concentration in the green beer.

OE also had a significant impact on the main fermentation time at a constant temperature. For example, the double increase in OE (from 8.53 °Р to 17.47 °Р) at 12.5 °C resulted in a 2.5-fold rise in the main fermentation time.

Raising the temperature to 15 °C led to trends similar to those observed in the fermentation at 10 °C. The threefold increase in *M*_IC_ caused a decrease in the primary fermentation time. The peaks of aldehydes and vicinal diketones were localized at the early stage of main fermentation. Thus, their reduction started during the main fermentation. Therefore, the beer maturation lasted between 4 and 7 days.

The last variant of Table 2 showed the shortest fermentation time. At this high temperature, the maximum amount of vicinal diketones and aldehydes was detected at 48 h, and their reduction required seven days. It is worth noting that the reduction started during the main fermentation.

### Modelling of maturation time

**Figure 5.**
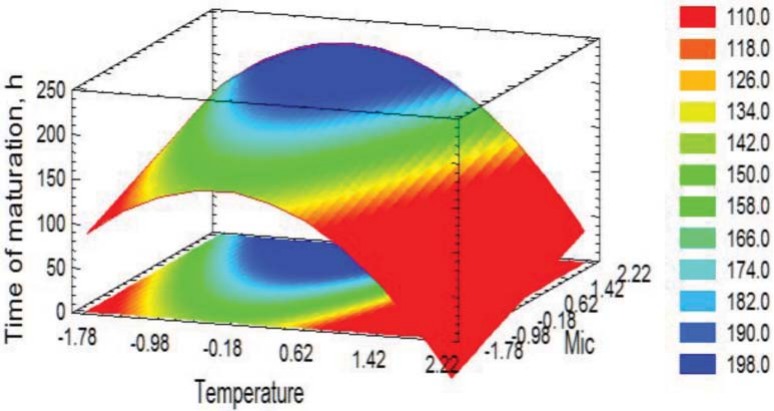
Estimated response surface for the influence of *T*_MAT_ = *T*_MF_ + 4 (A) and *M*_IC_ (B) on ‘Time of maturation’ at OE = 0 (13 °P). Factors were presented with coded values.

Similarly, a mathematical model was developed for the experimental data on maturation time shown in Figure 5 and Table S1 (see the Online Supplemental Appendix):(2) 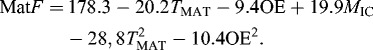

According to the model, the temperature had the most significant impact on the maturation time reduction. *M*_IC_ affected the objective function ‘negatively’. This can easily be explained by the fermentation dynamics. The higher *M*_IC_ and OE led to the synthesis of more carbonyl compounds, which resulted in increased maturation time (Figure 5). Nevertheless, Figure 5 illustrates an interesting phenomenon: there was a relatively small zone (between 11 and 13 °Р) wherein the increase in the extract did not lead to an increase in the carbonyl compounds concentration. This could be related to the provision of optimal growth conditions for the immobilized cells and the diffusion resistances in the system.

At the beginning of the maturation, a second peak of aldehydes was observed, which was essential for the model accuracy. The first peak appeared during the main fermentation (between 36 and 96 h). The second peak was observed between 12 and 24 h of maturation. The second peak was clearly defined for the higher extracts and its height was similar to the first one. Contrariwise, the lower extract led to indistinct peaks of aldehydes. The vicinal diketones maximum occurred during the main fermentation. The OE increase resulted in a peak displacement to a later period of the main fermentation. The vicinal diketones reduction started before the end of the main fermentation.

### Other changes during the fermentation

The pH and colour change during fermentation are very important for beer quality. The pH decreased by 0.5–0.6 units between 12 and 68 h depending on the operational conditions. Afterwards, the pH change was smooth. At the end of the fermentations, the pH values were typical of lager beer.

The beer colour decreased by 3–5 EBC units depending on the fermentation conditions. The colour of the beer produced by immobilized cells was darker than the traditional lager beer colour because of the wort sterilization before fermentation. It can be noted that the colour decreased further during the fermentation with the increase in *M*_IC_. This could be related to the increased surface area for the adsorption of the compounds forming the beer colour.

### Mathematical model optimization

The data on the model optimization (Equation 1) are presented in Table 3. The results allowed us to choose a range of suitable process parameters applicable to laboratory wort fermentation. In a series of experiments,[20] it was found that the real main fermentation time was 12 h longer than the main fermentation time determined by the model. This could be related to the system diffusion resistance which hindered the substrate transfer to the cells. Regarding the beer flavour profile, optimal results were achieved in variant 2 of Table 3. Although the experiments will continue with optimized variant 2, the other variants can also be used for lager beer production. Moreover, the data showed that overall the beer produced using all the tested variants was in the commercial product range.[20]

**Table 3.** Optimal values of the mathematical model ‘Time of main fermentation’ (Equation 1).

Time of main fermentation = 48 h				
Factor	Lower level	Upper level	Optimum	Real data
*T*_MF_	−1.78885	1.78885	1.28169	15.70 °C
OE	−1.78885	1.78885	−1.65848	8.85 °Р
*М*_IC_	−1.78885	1.78885	0.677704	13.38 g
Time of main fermentation = 60 h				
*T*_MF_	−1.78885	1.78885	1.0301	15.07 °C
OE	−1.78885	1.78885	−1.31274	9.72 °Р
*М*_IC_	−1.78885	1.78885	0.5428	12.71 g
Time of main fermentation = 72 h				
*T*_MF_	−1.78885	1.78885	1.0815	15.20 °C
OE	−1.78885	1.78885	−0.82211	10.94 °Р
*М*_IC_	−1.78885	1.78885	0.70442	13.52 g

The second mathematical model (Equation 2) can also be optimized. However, this is not necessary, since the model structure is related to the main fermentation model (Equation 1). Equation 2 can be used to determine the maturation time for the variants presented in Table 3. A combined optimization of the two models is also possible but this would make the interpretation of the results more complicated.

### System productivity

One of the purposes of the study was to select the fermentation conditions which would be transferred into a continuous fermentation system. According to [21], the empirical method was most suitable for the operational parameters transfer. According to it, the time constants determined should be kept permanent during the fermentation. The fermentation time could be regarded as a similar time constant but some difficulties were observed when only this parameter was used for the transfer.

A method of batch beer fermentation comparison was shown in [17]. According to this study, the leading parameter was system productivity, which could be estimated at various stages of fermentation and for various parameters: ethanol, green beer and beer. The productivity depended on the fermentation time and could be used for comparison between batch and continuous fermentation.

The ethanol productivity of the system (Figure S1 in the Online Supplemental Appendix) varied from 0.08 to 0.61 g dm^−3^ d^−1^, but for most of the variants it was in the range of 0.18–0.35 g dm^−3^ d^−1^. The highest productivity was observed when the main fermentation lasted 78 h (10.5 ºP, 15 ºC and 15 g immobilized cells). This could be related to the fermentation time reduction at low OE, high *T*_MF_ and high *M*_IC_. On the other hand, the OE increase led to the production of more ethanol.

The green beer productivity of the system depended on the ethanol productivity, the degree of fermentation and OE (Figure S1 in the Online Supplemental Appendix). The green beer productivity was in the range of 5.78–65.02 g dm^−3^ d^−1^, but the optimal variants had a productivity of approximately 20 g dm^−3^ d^−1^. The differences between beer productivity and green beer productivity were non-significant (Figure S1 in the Online Supplemental Appendix). The beer productivity varied between 8.26 and 69.61 g dm^−3^ d^−1^ and increased with the increase in OE and the optimal variants according to Table 3 had OE between 8.85 ºP and 10.94 ºP.

After a similar statistical analysis of the influence of various factors on green beer productivity, the following mathematical model was developed:(3) 
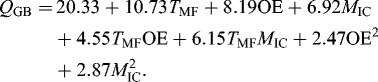



The green beer productivity can be used for the transfer of a batch fermentation to a continuous mode. Our subsequent research showed that the system productivity in a batch mode was related to the system productivity in a continuous mode. A fourfold increase in *M*_IC_ in the continuous fermentation resulted in a fourfold increase in the system productivity compared to batch fermentation.

## Conclusions

The influence of fermentation temperature, immobilized cell mass and original wort extract on beer fermentation with alginate-chitosan microcapsules with liquid core was investigated. The results showed that the factor that affected most significantly the fermentation time reduction was temperature. The increase in immobilized cell mass led to a reduction in the primary fermentation time but did not affect the total fermentation time. This was related to the synthesis of more carbonyl compounds, which caused prolonged maturation. The increase in the wort extract resulted in longer fermentation time. The model parameters were optimized and the data were used to produce three different beers under laboratory conditions. A mathematical model describing the system productivity was also developed. The data will be used for the transfer of the optimized fermentation conditions to a continuous beer fermentation system.
